# The role of RHAMM in cancer: Exposing novel therapeutic vulnerabilities

**DOI:** 10.3389/fonc.2022.982231

**Published:** 2022-08-10

**Authors:** Josephine A. Hinneh, Joanna L. Gillis, Nicole L. Moore, Lisa M. Butler, Margaret M. Centenera

**Affiliations:** ^1^South Australian Immunogenomics Cancer Institute and Adelaide Medical School, Adelaide, SA, Australia; ^2^Freemason’s Centre for Male Health and Wellbeing, The University of Adelaide, Adelaide, SA, Australia; ^3^Precision Cancer Medicine, South Australian Health and Medical Research Institute, Adelaide, SA, Australia; ^4^Nagoya University Graduate School of Medicine, Nagoya, Japan

**Keywords:** cancer, RHAMM, migration, signaling, microtubules

## Abstract

Receptor for hyaluronic acid-mediated motility (RHAMM) is a cell surface receptor for hyaluronic acid that is critical for cell migration and a cell cycle protein involved in microtubule assembly and stability. These functions of RHAMM are required for cellular stress responses and cell cycle progression but are also exploited by tumor cells for malignant progression and metastasis. RHAMM is often overexpressed in tumors and is an independent adverse prognostic factor for a number of cancers such as breast and prostate. Interestingly, pharmacological or genetic inhibition of RHAMM *in vitro* and *in vivo* ablates tumor invasiveness and metastatic spread, implicating RHAMM as a potential therapeutic target to restrict tumor growth and improve patient survival. However, RHAMM’s pro-tumor activity is dependent on its subcellular distribution, which complicates the design of RHAMM-directed therapies. An alternative approach is to identify downstream signaling pathways that mediate RHAMM-promoted tumor aggressiveness. Herein, we discuss the pro-tumoral roles of RHAMM and elucidate the corresponding regulators and signaling pathways mediating RHAMM downstream events, with a specific focus on strategies to target the RHAMM signaling network in cancer cells.

## Introduction

The development and progression of cancer is characterized by a number of hallmarks that include uncontrolled cellular proliferation and aberrant activation of the host tumor microenvironment (TME) (1). Cell proliferation is normally driven and regulated by the cell cycle machinery, and is essential for cell growth, DNA replication and cell division. However, this activity is usurped by tumor cells for uncontrolled replicative capacity and metastatic spread (2). In fact, genetic or pharmacological manipulation of proteins required for cell cycle progression is currently providing clinically-approved therapeutic targets to limit tumor growth (2, 3). The TME, comprising cellular (stroma, fibroblasts, endothelial cells and infiltrating immune cells) and non-cellular (extracellular matrix) components, is an important determinant of tumor fate and metastatic progression (4, 5). Multiple experimental and clinical studies have indicated that cancer cells control TME factors to facilitate disease progression, aggressiveness and drug resistance (4, 5). Of note, inhibition of components of the TME, typified by the success of anti-PDL1 immunotherapies, has become relevant for the treatment of cancer (6, 7). Accordingly, proteins with dual functions in both cancer cells and the TME are attractive therapeutic targets for cancer management.

One such protein is the Receptor for Hyaluronic Acid Mediated Motility (RHAMM). RHAMM has both extracellular and intracellular functions depending on its cellular location (8, 9). Extracellular RHAMM is a well-characterized hyaluronic acid (HA) receptor that modulates HA-induced cell migration critical for inflammation and wound healing (8, 10, 11). Intracellular RHAMM is a cell cycle protein that regulates mitotic spindle and microtubule formation (9, 12). Importantly, these physiological functions of RHAMM are often dysregulated in cancer for growth advantage and disease progression (11, 13). RHAMM expression in most homeostatic tissues is relatively low and its expression is induced upon inflammatory stimuli (11). In contrast, human tumors have been reported to overexpress extracellular RHAMM, and this overexpression is commonly associated with metastatic and aggressive phenotypes, as well as poorer disease outcomes in prostate, breast and hematological malignancies (13–16). RHAMM overexpression is also an independent prognostic factor in many cancers (17–19).

Given that RHAMM is chronically overexpressed in cancer, and has multiple functional roles in cancer development and progression, it has significant potential as a therapeutic target for cancer. Thus, the discovery of agents that interfere with the expression of RHAMM in tumors could provide novel therapeutic strategies to limit tumor growth. However, the current incomplete understanding of RHAMM functions and its varied cellular distribution restricts the development of effective RHAMM-targeted therapies. Herein, we (i) discuss the importance of RHAMM as a potential oncogene and (ii) examine the regulators and signaling pathways activated by RHAMM, with the aim of highlighting new therapeutic avenues.

## Structure and functions of RHAMM

**Figure 1:**
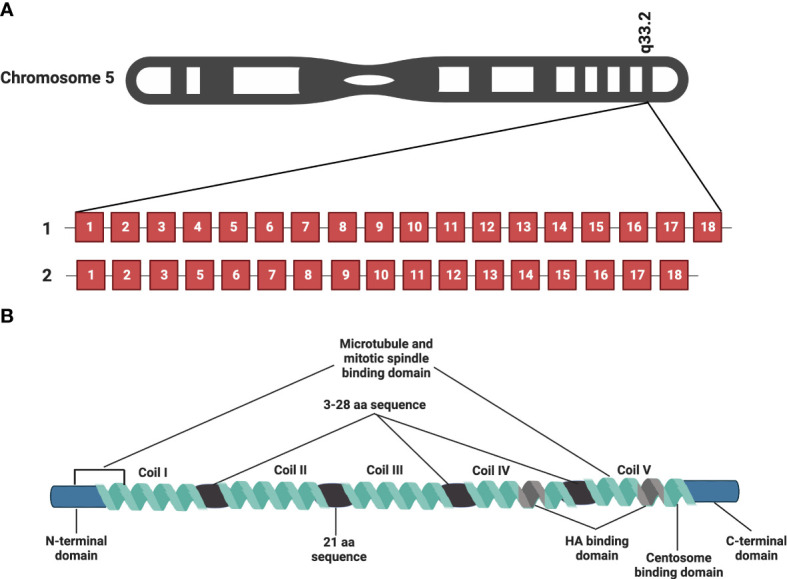
RHAMM gene and protein structure. *HMMR*, the gene encoding RHAMM is located on chromosome 5 and is made of 18 exons. **Figure 1A** is a representation of full-length *HMMR* and the most commonly occurring isoform, the exon 4 deficient variant (v3). **Figure 1B** is a diagrammatic representation of the protein structure of full length human RHAMM indicating the major functional domains. Five coils make up the supercoiled coiled domain with each separated by a 3-28 amino acid sequence with the exception of coil II and III, which are separated by a 21 amino acid sequence. The leucine-rich basic HA binding domain is located in coil IV and V close to the carboxyl terminal of the protein. Generated with BioRender.com.

RHAMM (also known as CD168) is a helical glycoprotein encoded by the gene *HMMR*, located on chromosome 5 (5q33.2-qter) in humans, with 18 exons and 2 start codons as depicted in **Figure 1A** (10, 20, 21). The full-length human RHAMM protein has a molecular weight of 84kDa and is made up of 725 amino acids (22, 23). Turley et al. first isolated and characterized RHAMM as part of the hyaluronic acid receptor complex (HARC) system from sub-confluent chick heart fibroblast cells (10, 24). Subsequent to this study, several RHAMM variants have been identified and are reported to occur *via* alternate gene splicing or the use of multiple start codons (10, 21, 23, 25). The best-characterized variant of RHAMM is the intracellular dominant exon 4 deficient variant (v3) (**Figure 1A**). RHAMM v3 is reported to localize with microtubules and induce oncogenic transformation when overexpressed in fibroblasts (23, 26). Additionally, RHAMM v3 is frequently detectable in cancer cells compared to normal tissues and has been shown to promote metastatic progression of tumors *in vivo* (23, 27). But why cancer cells express this particular variant is yet to be fully understood.

RHAMM is a supercoiled-coil hydrophilic protein with three main functional domains, namely the amino-terminal domain (about 163 amino acids), the rod-like middle supercoiled-coil domain (made up of 5 coils) and the carboxyl-terminal domain (about 11 amino acids) (10, 20, 22, 23). **Figure 1B** is a pictorial description of the RHAMM protein, identifying the key features and various functional domains. The supercoiled-coil domain is a feature responsible for mediating protein-protein interactions (12, 23, 28). RHAMM is known to interact with cell surface receptors such as CD44, and platelet derived growth factor receptor (PDGFR) to mediate cell migration (29–31). RHAMM also interacts with intracellular proteins such breast cancer gene 1 (BRCA1), targeting protein for Xklp2 (TPX2) and extracellular signal-regulated kinase 1/2 (ERK1/2) to regulate mitotic spindle formation and stability (28, 32, 33).

Despite being an HA-binding protein, RHAMM lacks the classic HA-binding motif (proteoglycan tandem repeat) and instead interacts with HA *via* a basic amino acid-rich domain denoted by B(X_7_)B, where B is a lysine or arginine and the X is a non-acidic amino acid (aa) located on aa635-646 and 657-666 (10, 20). This region has also been shown to overlap with a carboxyl basic leucine zipper motif responsible for the interaction with centrosomes during mitosis (12). The N-terminal domain (aa 40-59 and 76-90) and exon 4 encoded region localize with microtubules during interphase (12, 23, 26).

Physiologically, RHAMM is transiently upregulated in response to injury in fibroblast cells and it also promotes neural development by enhancing neuroprogenitor cell division (34–36). Consequently, *in vivo* studies in RHAMM null mice have demonstrated defective wound closure after injury and neurodevelopment abnormalities such as megalocephaly (34–37). In homeostatic adult human tissues, RHAMM is expressed in the testes, thymus and placenta, with very low expression levels in the lungs and pancreas (13, 38).

### Extracellular functions of RHAMM

RHAMM as an extracellularly expressed protein is predominantly in the lamellipodia at the leading edge of migrating cells (39, 40). As a well-characterized HA receptor, extracellular RHAMM modulates HA-induced cell migration (8, 10, 24). HA is an extracellular matrix (ECM) glycosaminoglycan, produced by hyaluronan synthases (HAS1-3) and undergoes fragmentation by chemical stress or enzymatic degradation to generate lower molecular weight fragments (11, 41). The size of the HA polymer is often predictive of its physiological function (11). The native high molecular weight HA, the most abundant in normal tissues, provides hydration and resistance to mechanical stress and further exhibits anti-migratory and anti-proliferative effects (11, 41). In contrast, low molecular weight HA (LMWHA), which preferentially interacts with RHAMM, promotes cell proliferation and migration in response to injury or tumor cell death (11, 41). Despite being extracellularly expressed, RHAMM lacks the signal peptide necessary for canonical ER-golgi export, hence it is speculated that RHAMM may be exported by some uncharacterized unconventional pathway(s) (42, 43). Surprisingly, no study to date has evaluated the exact mechanisms by which RHAMM is exported into the extracellular space, therefore studies focused in this direction may help unravel this aspect of RHAMM function and reveal therapeutic opportunities.

RHAMM lacks a transmembrane domain and therefore couples with a number of membrane-spanning receptors such as recepteur d’origine nantais (RON), CD44, PDGFR and transforming growth factor β1 (TGF-β1) to activate intracellular signaling pathways involved in cell migration (24, 29, 44, 45). For example, RHAMM couples with either CD44 in breast cancer cells or PDGFR in fibroblast cells to activate ERK1/2, in mediating cell migration (24, 29, 31, 44). HA and RHAMM interaction (HA : RHAMM) also activates other intracellular signaling pathways such as RHO-ROCK and β-catenin through other unknown cell surface partner proteins (46–51).

### Intracellular functions of RHAMM

RHAMM was first identified as a novel microtubule associated protein (MAP) that interacted directly with interphase and mitotic microtubules by Assman and co-workers using confocal microscopy, an observation later confirmed by Maxwell et al. (12, 23, 26). Similar to other MAPs, both full length and RHAMM v3 decorated the entire length of microtubules in multiple cell lines and were also found to localise with centrosomes and interact with other centrosomal proteins during mitosis (12, 23, 26, 28, 32). Functionally, RHAMM regulates mitotic spindle integrity and progression of cells through the G2/M phase of the cell cycle. Consequently, inhibition of RHAMM results in the formation of multipolar spindles and loss of spindle integrity whilst overexpression of RHAMM produces large centrosomes with a resultant metaphase block that leads to cell death (12, 26, 52).

**Figure 2:**
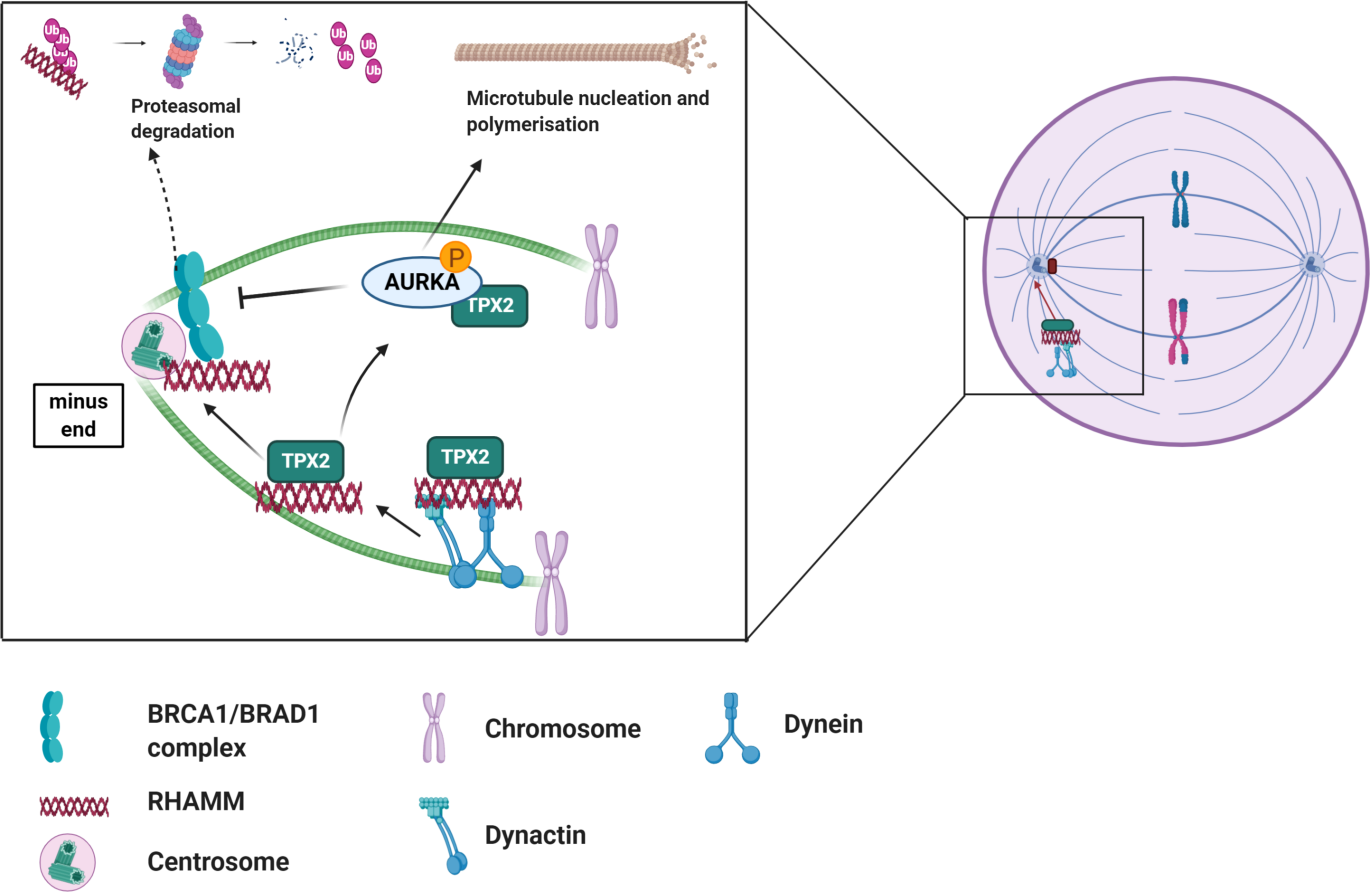
Overview of the role of RHAMM during cell cycle. RHAMM is a cell cycle gene localized at spindle assembly points where it associates with other MAPs such TPX2 to regulate spindle dynamics and microtubule stability. Like most cell cycle proteins, RHAMM mediated microtubule functions are regulated by BRCA1/BRAD1 complex which tags RHAMM for ubiquitination and subsequent proteasomal degradation, an event nullified by activation of AURKA. Generated with BioRender.com.

RHAMM interacts with a number of MAPS and centrosomal proteins in regulating microtubule dynamics. RHAMM binds to dynein motor complex, a cargo protein, during mitosis to correctly localize RHAMM to the minus end of spindle poles where it is involved in maintaining spindle stability by crosslinking centrosomal microtubules (**Figure 2**) (12, 52, 53). Additionally, RHAMM may contribute to microtubule formation indirectly by stabilizing and correctly positioning TPX2 for the effective activation of aurora kinase A (AURKA) at microtubule forming centers as depicted in **Figure 2** (28). During cell cycle progression, RHAMM is regulated by the BRCA1-BRAD1 E3 ubiquitin ligase system (54). RHAMM also acts as an adaptor protein to correctly position and direct ERK1/2 to mitotic spindle forming centers and this has been demonstrated to partly modulate RHAMM associated microtubule functions (12, 26, 55–58). Intracellular RHAMM also acts as a transcriptional co-activator to modulate the transcription of target genes involved in migration (59, 60). For example, RHAMM serves as a co-activator for the E2F1 transcription factor to mediate the transcription of fibronectin in melanoma cells (59).

Whilst RHAMM performs disparate extracellular and intracellular roles, it is becoming more apparent that these roles may be somewhat linked. The observation that HA expression increases during mitosis and the ability of exogenous HA to modify RHAMM : TPX2 binding affinity and alter AURKA activity, further demonstrates that HA : RHAMM interactions may contribute in part to RHAMM intracellular functions (61, 62). A detailed understanding of the RHAMM interactome may yield further insights into the factors linking its intra- versus extracellular actions.

## Molecular signaling pathways regulated by RHAMM

Interaction between HA : RHAMM is known to regulate a number of downstream signaling pathways; the most predominant are the ERK1/2 and RHO-ROCK pathways (29, 31, 36, 63). It was first reported that H-ras transformed cells exhibited characteristics similar to RHAMM overexpressing cells, and that loss-of-function mutation of RHAMM resulted in decreased ERK1/2 activation in Ras mutant cells (31, 64). Subsequent studies demonstrated that RHAMM overexpressing cells exhibit high basal ERK1/2 activation while RHAMM null fibroblasts showed reduced ERK1/2 phosphorylation with no change in total ERK1/2 levels (31, 36). RHAMM has been shown to co-localize with ERK1/2 in breast cancer cells, but the exact mechanism by which RHAMM activates ERK1/2 is currently unknown (29).

However, studies have suggested that RHAMM may activate ERK1/2 pathways by partnering with a number of cell surface proteins in response to HA stimulation (26, 62). RHAMM has been shown to co-localize with CD44, another well-characterized receptor for HA, in aggressive MDA-MB-231 breast cancer cells to maintain the activity of ERK1/2 (29). Additionally, RHAMM may co-activate ERK1/2 by partnering with upstream activators such as PDGFR and epidermal growth factor receptor (EGFR) in mesenchymal and fibroma cells respectively to promote cell motility (36, 62). Once activated, ERK1/2 facilitates cell motility by either activating the focal adhesion pathway (FAK) pathway or by mediating transcription of motogenic genes such as MMP9 in a RHAMM dependent manner (46, 47, 60).

HA : RHAMM interaction is also reported to activate the RHO-ROCK signaling pathway in prostate cancer cells, ultimately leading to the phosphorylation of translation initiation factor eIF4E, an observation that was associated with treatment resistance (65). Functionally, HA : RHAMM interaction promoted cell proliferation, metastasis and invasion of androgen independent PC3 prostate cancer cells, and this observation was abrogated by a ROCK inhibitor (50, 65).

**Figure 3:**
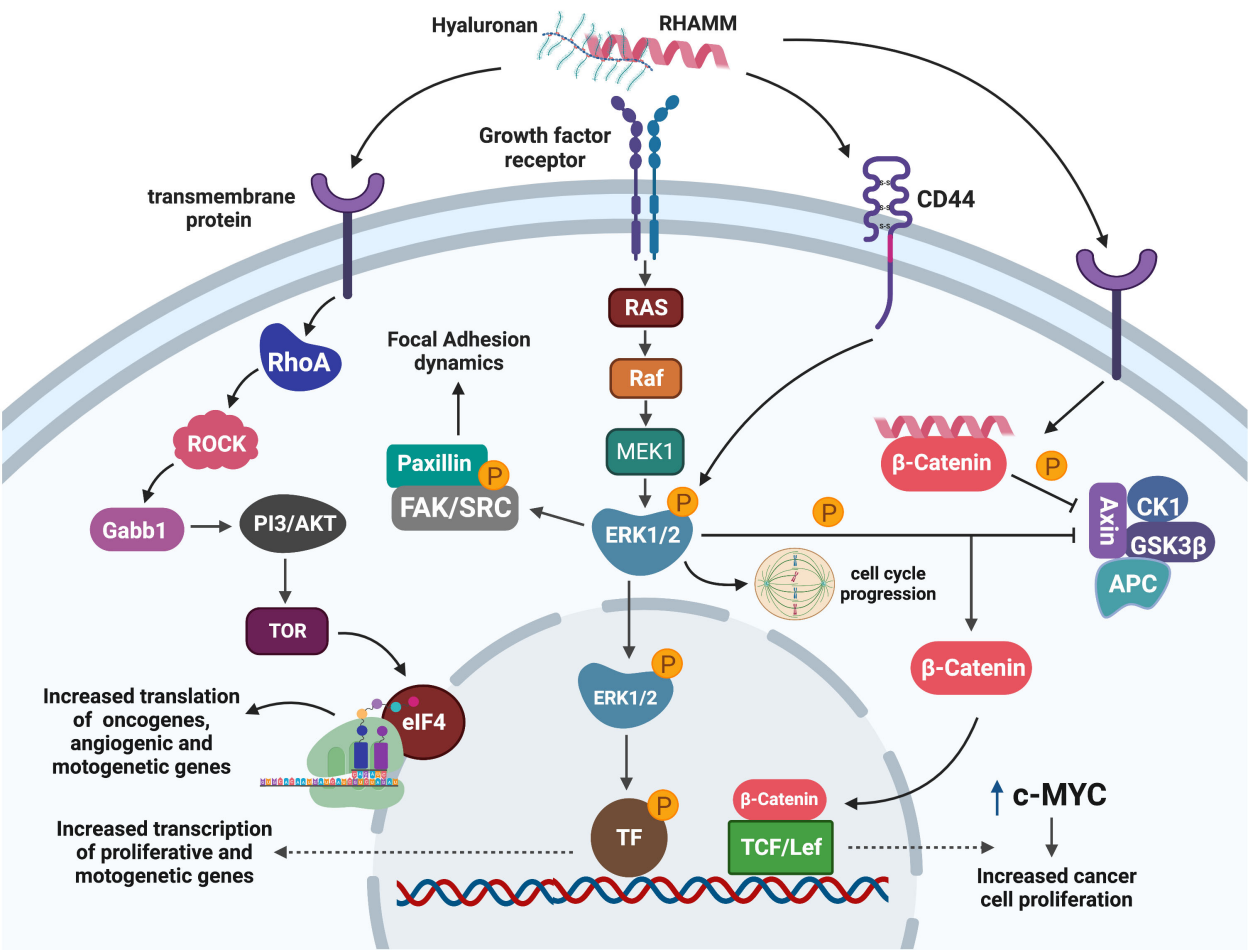
Signaling pathways mediating RHAMM oncogenic effects. Cell surface HA : RHAMM interaction may co-localize with transmembrane receptors/proteins to activate intracellular signaling pathways that result in the phosphorylation of ERK1/2, the key RHAMM modulated protein. pERK1/2 influences cell migration by regulating cell adhesion dynamics through FAK or is translocated into the nucleus to activate unknown transcription factors that enhance the transcription of mitogenic and motogenic genes. Alternatively, pERK1/2 may influence mitotic entry by regulating mitotic spindle formation needed for effective progression through mitosis. Activation of RHAMM may also result in the translation of growth-promoting and motogenic genes through the ROCK-eIF4E pathways. Similarly, RHAMM augments the stability of β-catenin by inhibiting the β -catenin degradation complex and hence promotes nuclear translocation and activation of transcription factors which enhance transcription of the oncogene c-MYC with a resultant increase in cell proliferation. Generated with BioRender.com.

Stimulation of RHAMM by HA has also been shown to activate less reported pathways such as β-catenin to up-regulate the expression of the oncogene c-MYC which promoted tumour growth and proliferation in fibrosarcoma cells (51). **Figure 3** is a summary of the RHAMM signaling pathway.

## Role of RHAMM in oncogenesis

RHAMM plays an important role in the development and progression of a number of cancers (11). The oncogenic potential of RHAMM is attributed to its intracellular function in cell cycle progression, specifically mitotic spindle formation and stability and extracellular functions in cell migration. In cancer, RHAMM overexpression has been reported in breast, prostate, leukemia, pancreatic, lung cancers, and glioblastoma, with strongest expression in metastatic tumors (38, 64, 66–71). The significance of RHAMM in cancer is documented below and tabulated in **Table 1** is a summary of the role of RHAMM reported in various malignancies.

**Table 1:** A summary of the oncogenic role of RHAMM in various cancers.

Cancer type	Expression(Protein, RNA)	Major findings	Associated Pathways	References
Breast Cancer	overexpressed	Highly expressed in metastatic tumors. Overexpression is associated with poorer overall, disease free and metastatic free survival. Linked with increased tumor grade. RHAMM overexpression increased migration of breast cancer cells. Associated with increased breast cancer risk in BRCA1 mutation carriers	MAPK (ERK1/2) Pathway Mevalonate-Hippo pathway	(29, 32, 33, 48, 66, 67, 72, 73)
Hematological cancers	overexpressed	Overexpressed in Chronic myeloid leukemia (CML), acute myeloid leukemia (AML) and multiple myeloma (MM). Overexpression of full length and v3 are associated with poorer overall survival and disease progression but more pronounced with v3. RHAMM was identified as an antigenic target with subsequent development of RHAMM-R3 peptide for phase I and II clinical trials.		(16, 38, 52, 74–77)
Prostate Cancer	overexpressed	Overexpression is associated with biochemical recurrence, increased Gleason score and poorer prognosis. RHAMM expression is increased in metastatic tumors and mediates cell migration in prostate cancer cells. Androgen and Retinoblastoma regulated gene. RHAMM activates ROCK pathway to promote the development of castration resistance. RHAMM expression increases with the duration of ADT therapy. Potential biomarker for disease progression. *HMMR* is a hypoxia induced gene.	ROCK-eIF4E pathway	(15, 19, 49, 65, 68, 78, 79)
Lung Cancer	overexpressed	Overexpression is with associated decreased overall survival, poorer disease outcome and disease progression. Increased expression in metastatic tumors and cell lines. *HMMR* knockdown increased miR34a (a tumor suppressor) expression and prevented establishment of H2030-BrM3 lung cancer cells *in vivo*		(18, 69, 80–85)
Bladder Cancer	overexpressed	Overexpression is with associated with poorer disease specific and overall survival and increased mortality. Higher expression in invasive and higher grade tumors. In a UPII-SV40Tag progressive bladder cancer model, *HMMR* was associated with early stage bladder cancer development. *HMMR* knockdown resulted in decreased proliferation in bladder cancer tumor xenograft model.		(86–89)
Colorectal Cancer	overexpressed	Independent prognostic marker. Poorer overall survival Overexpression is associated with aggressive and metastatic tumor phenotypes. *HMMR* knockdown in J82 colorectal cancer cell line decreased cell proliferation, invasion and metastasis *in vitro* and *in vivo* and this was associated with G2/M cell cycle arrest	MAPK (ERK1/2)	(17, 90–93)
Pancreatic Cancer	overexpressed	Independent prognostic marker. Associated with poorer overall survival Full length and RHAMM variant 3 (RHAMM B) were overexpressed in metastatic tumors with RHAMM B being more expressed. *HMMR* B corresponded with worse prognosis. RHAMM B overexpression increased metastasis of BON1-TGL pancreatic neuroendocrine cells *in vivo* whilst full length RHAMM had no effect. EGFR activation was partially responsible for RHAMM B mediated metastasis *in vivo* *HMMR* knockout generates RHAMM (exon 8-16) deficient variant which promotes PDAC invasiveness.	EGFR	(25, 27, 70)
Glioblastoma	overexpressed	Upregulated in Glioblastoma stem cells GSC relative neural stem cells. Associated with poorer overall survival in mesenchymal glioblastoma patients. Knockdown of *HMMR* repressed proliferation of GSC and reduced their ability to form neurospheres. Downregulation of *HMMR* reduced the expression of stem cell markers and transcription factors. *HMMR* knockdown in GSC cells had reduced ability to form tumors compared to *HMMR* competent cells *in vivo*.		(71, 94, 95)

### Role of RHAMM in cell proliferation

The critical role of RHAMM in the cell cycle means that overexpression or downregulation of RHAMM results in the accumulation of cells at G2/M phase (67, 96–98). Levels of RHAMM mRNA and protein have both been reported to increase during G2/M phase, but the exact mechanism mediating transcriptional upregulation of RHAMM during cell cycle progression remains unknown (98). Multiple independent studies have reported that downregulation of RHAMM suppresses cell proliferation in cancer cells and that overexpression of RHAMM is transforming (64, 97, 99). One mechanism by which RHAMM may promote cell proliferation is by augmenting the activity of M phase promoting factor Cdc2/cyclin B currently known as the Cdk1/cyclin B kinase activity through stabilisation of Cdc2 mRNA levels in an HA dependent manner (80, 97). Cdk1/cyclin B complex regulates mitotic entry by phosphorylating a number of spindle associated proteins such as importin to control effective microtubule formation and chromosomal segregation (100). Therefore, RHAMM overexpression may facilitate premature mitotic entry resulting in genetic alterations that favour tumour growth through an increase in Cdk1/cyclin B activity.

In regulating cell cycle progression, RHAMM is reported to associate with other MAPs to regulate mitotic spindle assembly. RHAMM interacts with TPX2 during mitosis to correctly localize TPX2 to the minus end of microtubules for AURKA phosphorylation and activation (28, 101). Activation of AURKA promotes centrosome nucleation and maturation for mitotic progression (28). Cancer cells overexpressing RHAMM exhibit increased AURKA activation, hence increased mitotic progression and proliferation. As a result, knockdown of RHAMM increased the sensitivity of malignant peripheral nerve sheath tumors to the AURKA inhibitor MLN8237 (102).

Similar to most cell cycle proteins, RHAMM activity is regulated by the BRCA1-BRAD1 E3 ubiquitin ligase complex, which targets it for degradation during cell cycle progression (54). Thus, RHAMM overexpression (in BRCA1 competent cells) has been shown to induce phenotypic changes similar to BRCA1 deficient cells, which are characterized by polyploidy, formation of micronuclei, centrosomal amplification and chromosomal mis-segregation; genomic events associated with DNA damage and mutations (72). In the event of RHAMM overexpression, which is indeed the case for most cancers, cancer cells may mimic BRCA1 deficient phenotypes that facilitate genomic instability and hence promote tumorigenesis (32, 72). Additionally, overexpression of RHAMM is linked to increased AURKA activation, which corresponds to decreased BRCA1 ubiquitin activity, thereby providing a BCRA1 deficient environment necessary for genomic instability (103). A more recent study has demonstrated that loss of BRCA1 and overexpression of *HMMR* promotes AURKA activation which results in ARPC2-mediated decreased mitotic cortex stability and subsequently genomic instability (104). Thus, RHAMM has been linked with increased breast cancer susceptibility in BRCA1 mutation carriers (32, 67, 104).

### Role of RHAMM in cell migration/metastasis

RHAMM influences cell motility in two ways, first by influencing mechanical changes in the cell that impacts unidirectional movements, and second by enhancing epithelial-to-mesenchymal transition (EMT). These functions are linked to RHAMM’s extracellular activity, which is achieved through coupling with transmembrane protein partners to activate intracellular signaling pathways, particularly ERK1/2 (29, 31). The role of ERK1/2 in cell motility has been well documented over the years and reviewed by Tanimura et al. (105).

Relevant to RHAMM is that ERK1/2 regulates the association between FAK and paxillin, a focal adhesion (FA) adaptor protein, by ensuring a phosphorylation/dephosphorylation feedback loop that influences FA assembly and disassembly during cell migration (46, 106, 107). Activation of the ERK1/2 pathway by HA : RHAMM interaction increased tyrosine phosphorylation of FAK with a resultant increase in FA turnover and consequently cell migration (46, 47). In regulating FAK signaling, RHAMM has also been reported to co-activate the E2F1 transcription factor to increase the transcription of fibronectin, which promoted integrin-β1-FAK induced motility in melanoma cells (59). RHAMM induced ERK1/2 activation also exerts control on mechanotaxis by regulating the activity of proteins involved in protrusion and matrix adhesion, therefore aiding detachment and polarized movement (105).

In terms of EMT, RHAMM mediated ERK1/2 activation facilitates that nuclear translocation and activation of yet to be identified transcription factors that mediate the transcription of EMT genes such as MMP9 (60), hence promoting the development of the mesenchymal phenotype (105). Indeed, inhibition of RHAMM using a mimetic peptide, P15-1, coincided with decreased myofibroblast differentiation and expression of mesenchymal markers such as vimentin and N-cadherin, resulting in decreased migration of fibroblast and prostate cancer cells (108). Similarly, inhibition of HA : RHAMM interaction suppresses TGF-β1 (a potent regulator of EMT) mediated cell migration (45, 109, 110). RHAMM-mediated decreased TGF-β1 activity was associated with decreased mesenchymal transformation and ultimately a decrease in cell migration (45, 108). It is still quite unclear how RHAMM modulates TGF-β1 induced cell migration and therefore future studies focused on understanding this phenomenon would provide better insights for effective targeting of this pathway.

HA : RHAMM interactions can also influence cytoskeletal changes of migratory cells by activating the RHO-ROCK pathway to mediate actin polymerization and promotion of unidirectional movement at the leading edge of the cell (50, 65, 111, 112). Overexpression of RHAMM in prostate cancer cells increased phosphorylation of ROCKII and coflin with an accompanying increase in filopodia formation and EMT markers, vimentin and N-cadherin, and a decrease in E- cadherin (50). The RHAMM-ROCK pathway is commonly hyperactivated in castration-resistant prostate cancer and associated with metastatic disease progression (50, 65). Collectively, these findings suggest a critical role for RHAMM in cell migration and more importantly metastasis of cancer cells and further underscores the relevance of targeting RHAMM in advanced cancers.

## Regulators of RHAMM expression

**Figure 4:**
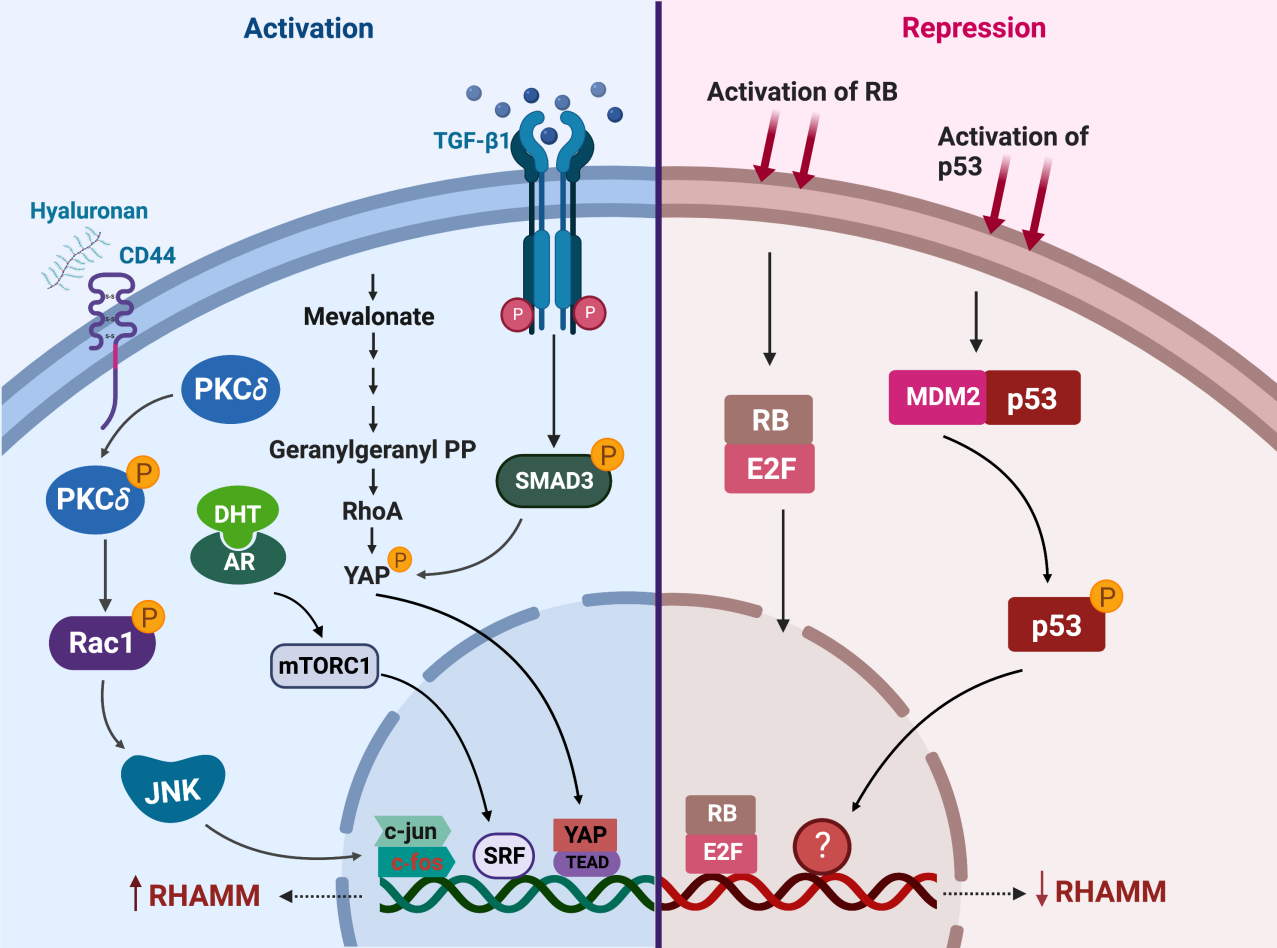
Regulators of RHAMM expression. This figure enumerates the transcriptional regulators of RHAMM activation (on the left) and repression (on the right) in cancer and normal cells. Activation of CD44, mevalonate, TGF-β1 or the androgen receptor (AR) signaling pathways induces transcriptional upregulation of *HMMR*. On the contrary, p53 and RB activation results in decreased transcription of *HMMR*. Generated with BioRender.com.

RHAMM is transcriptionally regulated by a number of factors that either activate or repress its expression (**Figure 4**). HA is a potent transcriptional up-regulator of RHAMM in both normal and transformed cells (36, 113, 114). Expression of *HMMR* was significantly increased when fibroblast, HUVEC and breast cells were stimulated with HA (45, 114, 115). Mechanistically, HA induced RHAMM gene and protein expression by activating CD44/protein kinase Cδ (PKCδ) pathway (115). Activation of the CD44/PKCδ complex induced c-fos and c-jun nuclear translocation, resulting in increased RHAMM transcription, an effect rescued by the inhibition of PKCδ (115).

Stimulation of fibroblasts with TGF-β1 markedly increased both *HMMR* transcription and cell surface expression of RHAMM (45, 109). TGF-β1-induced RHAMM transcription was mediated through a SMAD3-YAP dependent pathway (110). The increase in RHAMM expression enhanced TGF-β1-induced cell migration, and this effect was partially ablated by the addition of RHAMM blocking antibodies (45, 110, 115). Also mediated *via* YAP, but through prenylation of RhoA, mevalonate treatment of breast cancer cells induced both RHAMM protein and mRNA expression (48). These observations were nullified by the inhibition of YAP or treatment with simvastatin, a mevalonate inhibitor, and this consequently resulted in decreased cell migration in an ERK1/2 driven manner (48).

RHAMM is also transcriptionally up-regulated by DHT treatment in LNCaP prostate cancer cells (49, 116). Whilst the exact mechanism (s) underpinning DHT-induced RHAMM expression is currently unknown, it was reported that this could be a DHT-mediated mTORC1 activation of the SRF transcription factor which directly binds to the promoter region of *HMMR* in LNCaP cells (116). Further studies are required to fully clarify the direct association between the androgen receptor (AR) and mTOR pathways in regulating *HMMR* expression given that crosstalk between mTOR and AR is complex and context-dependent (117, 118).

Transcriptional downregulation of RHAMM has been achieved through activation of tumor suppressors p53 and retinoblastoma (RB) (50, 98, 119). Induction of p53 in D53wt ovarian cancer derived cells significantly repressed both full length and v3 RHAMM gene and protein expressions, likely through an indirect mechanism since no putative p53 binding site was observed on the *HMMR* gene transcript (98). Further studies are needed to provide insights into how p53 represses *HMMR* expression transcriptionally during cell cycle progression. Similarly, RB regulates RHAMM gene and protein expressions through the E2F1 transcription factor, which binds directly to the promoter region of RHAMM as observed in prostate and lung cancer cells (50). RNA sequencing of RB deficient cells identified *HMMR* as one of the most significantly upregulated genes promoting cell migration in response to RB-loss (50). Accordingly, drugs that induce or augment p53 or RB activity such as Nutlin-3 and CDK4/CDK6 inhibitors respectively, repressed RHAMM expression (50, 98).

## Therapeutic approaches to target RHAMM

### Immunological targeting of RHAMM

RHAMM was first identified as a tumor associated antigen in multiple myeloma and chronic myeloid leukemia (CML) and that overexpression was associated with poor prognosis in these patients (16, 38). This discovery stimulated preclinical immunogenic targeting of RHAMM in mouse glioma and melanoma models wherein mice received either dendritic cells pulsed with *HMMR* mRNA or a DNA-based xenopus RHAMM (xRHAMM), respectively (120, 121). In both studies, RHAMM vaccination significantly reduced tumor burden and increased infiltration of RHAMM specific cytotoxic T cells (120, 121). Following these discoveries, the RHAMM-R3 peptide vaccine was developed and evaluated in Phase I and II clinical trials (122, 123). In the first study, six chronic lymphocytic leukemia patients were vaccinated and about 80% of the patients produced significant numbers of CD8+ T cells with a 20% decrease in white blood cells (122). To further expand the therapeutic scope of the peptide vaccine, Greiner in that same year evaluated a high dose of RHAMM-R3 peptide in patients with acute myeloid leukemia (AML), multiple myeloma and myelodysplastic syndrome, where 4 out of nine patients showed an increase in CD8+ T cells and 3 showed positive clinical response (74). It was later reported that the RHAMM-R3 vaccine when co-administered with chemotherapy or stem cell transplantation in leukemic patients may delay relapse and ensure patients remained in remission (123).

Despite these promising initial clinical reports, RHAMM-R3 peptide is yet to receive clinical approval. To understand the therapeutic limitations of RHAMM-R3 peptides, Snauwaert and co-workers showed that for AML patients, RHAMM expression on normal hematopoietic cells were similar to that of leukemic stem cells and that expression was cell cycle dependent. Thus, they proposed that the lack of specificity of RHAMM expression limits its therapeutic application in AML (124). But for reasons not unique to the RHAMM vaccine, cancer vaccine development in general has encountered substantial setbacks due to immune tolerance and immunosuppressive factors in the tumor microenvironment (125, 126). A potential alternative approach would be to combine RHAMM vaccines with immune checkpoint inhibitors as a mechanism to evade these immunosuppressive factors (127).

### Targeting RHAMM signaling

The dependence of extracellular RHAMM on HA for effective signaling coupled with the oncogenic nature of LMWHA (RHAMM specific HA fragments), proffers HA as an effective target for limiting RHAMM downstream signaling activities.

Using phage technology, Tolg and colleagues first identified a number of HA binding peptides, worthy of note is Peptide 15-1 (P15-1), which exhibited substantial anti-migratory effects on fibroblasts in a dose-dependent manner and specifically served as a decoy for LMWHA (108). Subsequent to this discovery, these peptides have been evaluated in a number of preclinical cancer models and have shown significant effects on proliferation, angiogenesis, metastasis and invasion (15, 50, 108, 128). Mechanistically, P15 treatment markedly reduced the expression of TGF-β1, and suppressed the FAK pathway as identified in a pathway analysis from a microarray study of P15-1 treated fibroblasts (108). Additionally, a number of tubulin derived HA targeting peptides have also been developed against RHAMM-specific HA activities (129). A unique feature of these peptides was that they were internalised by breast and prostate cancer cells, suggestive of an inherent ability of these peptides to possibly interfere with RHAMM’s intracellular functions, although this requires experimental substantiation (129). These peptides present a burgeoning area of research worth exploring for the effective development of RHAMM specific anticancer therapies. However, the clinical use of peptides as therapeutics are limited by their unfavourable physicochemical properties (130). Whilst the identification of specific RHAMM therapies have mainly focused on identifying peptides that interfere with RHAMM function, their application have been restricted to *in vitro* assays with limited *in vivo* efficacy studies. Currently, the use of peptidomimetics (compounds that mimic a natural peptide in their ability to bind to specific receptors but with improved physicochemical properties) are becoming widely used alternatives in clinical therapeutics (131). Therefore, future studies focused on identifying and evaluating RHAMM peptidomimetics may accelerate the *in vivo* use of these peptides and potentially facilitate its clinical application.

Alternatively, therapeutic approaches focused on inhibiting HA synthesis 4-Methylubelliferone (4-MU) or HA fragmentation by hyaluronidase (O-sulphated HA) have been explored as anti-cancer therapies (132–134). These HA targeted therapies additionally downregulate RHAMM expression, making them attractive alternatives for modulating RHAMM signaling. These pharmacological agents regulate cancer promoting pathways such PI3K/AKT, EGFR and ERK1/2/MAPK to promote apoptosis and repress proliferation, migration, angiogenesis and invasion of cancer cells (132–134). 4-MU is a clinically approved drug sold under the brand name Hymercromone for use as an anti-spasmodic on the biliary tract in Europe and some parts of Asia (135). Despite multiple studies extensively showing the anti-cancer effects of 4-MU, it is yet to be evaluated in any clinical trials for its anti-cancer effects.

## Conclusion and future directions

There has been a growing appreciation of the role of RHAMM in cancer over the last three decades. A plethora of recent studies, incorporating cell and animal models as well as patient samples, have delved into the mechanisms associated with the individual identified roles of RHAMM in an attempt to understand the significance of this protein in cancer. More importantly, efforts have been made to explore the therapeutic potential of targeting RHAMM and to this end, much progress has been made with respect to preclinical evaluations, but the translation of these observations into patients has not yet materialised.

To fully expedite the translation of all these preclinical data into clinical use, it will first of all be important to clearly define the diverse compartmental functions of RHAMM. Whilst a lot of information exists on the intracellular and extracellular functions of RHAMM, much more insight is needed to decipher whether these functions are independent of each other or if there is some form of crosstalk between these identified roles. By employing computational biology approaches to existing transcriptomic and proteomic data, a RHAMM interactome can be developed to identify all RHAMM associated genes and proteins involved in cell cycle and migration and how these proteins are interconnected. This may also help identify functional nodes within these networks which are targetable by drugs. Additionally, different variants of RHAMM have been identified and are reported to play functional roles in oncogenic transformation, but current knowledge about how these isoforms are generated and their specific roles in RHAMM-mediated cancer development and progression are yet to be fully stratified. More *in vitro* and *in vivo* studies should be conducted to identify the mechanisms of generating these isoforms and with the aid of omics, proteins and genes associated with these isoforms may be identified to subsequently provide more insight into which RHAMM specific role they regulate.

It has been reported that RHAMM is exported to the extracellular space by yet to be identified mechanisms, where it associates with transmembrane proteins to activate intracellular signaling pathways. Shedding light on these mechanism(s) may provide new therapeutic targets to block the extracellular functions of RHAMM, hence providing treatment alternatives for the management of advanced disease. Also, RHAMM co-localizes with other transmembrane proteins to activate intracellular signaling pathways due to the lack of a transmembrane domain. Although some transmembrane partners have been identified, there are a number yet to be discovered, and even for the identified cell surface partners, the mechanism by which RHAMM modifies their activity is largely unknown. Therefore, identifying and further clarifying these interactions using affinity purification and *in silico* protein-protein interaction models, may provide mechanistic insights and also reveal indirect therapeutic targets for cancer and other inflammatory related diseases.

Taken together, RHAMM is a vital driver of cancer progression and metastasis, and by addressing some of the knowledge gaps identified in this review article, more specific and effective RHAMM-targeted therapies can be developed for the management of cancer.

## Author contributions

Study conception and design, JH, NM, JG, LB, and MC. Drafting of manuscript, JH, NM, and JG. Critical review of the manuscript, LB and MC. All authors contributed to the article and approved the submitted version.

## Funding

This research was funded by a grant from Cancer Australia (Project Grant AP1138766). MC was supported by the Freemasons Centre for Male Health and Wellbeing. LB was supported by a Beat Cancer SA Beat Cancer Project Principal Cancer Research Fellowship (PRF1117). JAH is supported by the Beacon of Enlightenment Scholarship from the University of Adelaide

## Conflict of interest

The authors declare that the research was conducted in the absence of any commercial or financial relationships that could be construed as a potential conflict of interest.

## Publisher’s note

All claims expressed in this article are solely those of the authors and do not necessarily represent those of their affiliated organizations, or those of the publisher, the editors and the reviewers. Any product that may be evaluated in this article, or claim that may be made by its manufacturer, is not guaranteed or endorsed by the publisher.
